# Broadband Quantum Dot Superluminescent Diode with Simultaneous Three-State Emission

**DOI:** 10.3390/nano12091431

**Published:** 2022-04-22

**Authors:** Cheng Jiang, Hongpei Wang, Hongmei Chen, Hao Dai, Ziyang Zhang, Xiaohui Li, Zhonghui Yao

**Affiliations:** 1 School of Nano-Tech and Nano-Bionics, University of Science and Technology of China, Hefei 230026, China; j1548625127@163.com (C.J.); hpwang@mail.ustc.edu.cn (H.W.); 2 School of Electronic and Information Engineering, Qingdao University, Qingdao 266071, China; daihao0117@163.com; 3 Qingdao Yichen Leishuo Technology Co., Ltd., Qingdao 266000, China; mamie_chm@ycleishuo.com; 4 School of Physics & Information Technology, Shaanxi Normal University, Xi’an 710119, China

**Keywords:** molecular beam epitaxy, quantum dots, superluminescent light-emitting diodes, optical coherence tomography, excited states

## Abstract

Semiconductor superluminescent light-emitting diodes (SLEDs) have emerged as ideal and vital broadband light sources with extensive applications, such as optical fiber-based sensors, biomedical sensing/imaging, wavelength-division multiplexing system testing and optoelectronic systems, etc. Self-assembled quantum dots (SAQDs) are very promising candidates for the realization of broadband SLED due to their intrinsic large inhomogeneous spectral broadening. Introducing excited states (ESs) emission could further increase the spectral bandwidth. However, almost all QD-based SLEDs are limited to the ground state (GS) or GS and first excited state (ES_1_) emission. In this work, multiple five-QD-layer structures with large dot size inhomogeneous distribution were grown by optimizing the molecular beam epitaxy (MBE) growth conditions. Based on that, with the assistance of a carefully designed mirror-coating process to accurately control the cavity mirror loss of GS and ESs, respectively, a broadband QD-SLED with three simultaneous states of GS, ES_1_ and second excited-state (ES_2_) emission has been realized, exhibiting a large spectral width of 91 nm with a small spectral dip of 1.3 dB and a high continuous wave (CW) output power of 40 mW. These results pave the way for a new fabrication technique for high-performance QD-based low-coherent light sources.

## 1. Introduction

Superluminescent light-emitting diodes (SLEDs), which combine a high brightness of semiconductor lasers and a broadband emission spectrum of semiconductor light-emitting diodes, have emerged as ideal low-coherence light sources for a number of applications, such as optical fiber-based sensors, biomedical imaging, wavelength division multiplexing system testing, and optoelectronic systems, etc. [1,2,3,4]. In particular, SLEDs have been recognized as one of the most important light sources in the near-infrared (NIR) and mid-infrared (MIR) regimes for optical coherence tomography (OCT) techniques to achieve high axial resolution images of biomedical samples for clinical applications [5,6,7].

Among those applications, it is desirable to have both high-power output and broadband emission at the same time. However, there is always a trade-off between high-power output and broadband light emission with current commercial quantum-well-based SLEDs [8]. Various methods have been applied to enhance the performance of quantum-well superluminescent light-emitting diodes (QW-SLEDs), such as using chirped QW structures or incorporating the emission from higher-order transitions of a QW; however, carrier distribution and photon reabsorption in asymmetric multi-QW structures are still issues for the above devices [9].

It is well-known that SAQDs constructed by the Stranski–Krastanov (S-K) epitaxy growth mode have been studied extensively for making high-performance lasers over the past three decades [10,11,12,13,14]. As carriers in the QDs structures are strongly confined in all three-dimensional space, the thermal distribution in the QD structure is much smaller than that in bulk and QW structures, which allows one to obtain a low threshold current, high wavelength stability under direct modulation, and high temperature insensitivity for a QD laser [15,16,17]. The fabrication of QD based lasers usually demands a high uniform dot size distribution to achieve a high peak gain, but the large dot size fluctuation cannot be completely avoided for the QDs grown by the S-K growth mode. Conversely, the nonuniform dot size distribution results in a large inhomogeneous broadening of the QD ensemble, which can be used to gain a naturally broad spectral emission, so SAQDs materials have attracted significant attention as a promising means to realize broadband SLEDs [18,19,20]. SAQDs have an approximately Gaussian distribution in size, shape, and composition, which are very important to obtain a symmetric gain spectrum for device applications. In addition, SLEDs based on QDs have the potential to achieve a large saturated output power due to the presence of a large carrier reservoir in the ESs and the wetting layer (WL) of the QDs. More importantly, the light emission from GS of QDs can reach its saturation power under low injection currents due to the low GS saturation gain of the QDs, which leads to the light emission from the ES_1_ of QDs occurring easily when compared to QW-SLEDs [21,22,23]. This feature is very promising in the manufacturing of an SLED with simultaneous high power and broad bandwidth.

To satisfy high power and broad spectral bandwidth in a QD-SLED, a variety of methods have been reported. A high-power QD-SLED with over 200 mW output has been achieved using a multiple five-QD-layer structure at 1 µm wavelength range [11]. Following the above work, a large dot size distribution could be acquired using a high growth rate or a low growth temperature, which is desirable to fabricate a broadband QD-SLED [19]. Subsequently, introducing a chirped multiple-QD-layer structure by changing the In% in the InGaAs capping layer of each InAs QD layer as the gain medium, a 85 nm broadband QD-SLED has been created [24]. The chirped QD structure can also be realized by changing the dot size in each QD-layer structure. In addition to the epitaxy growth, broadening the emission spectra of a QD-SLED could also be achieved using the post-growth rapid thermal annealing (RTA) technique, using a laser annealing process [4,25], combining patterning techniques [26], or using the laser annealing process to realize selective area intermixing [27]. To have a high gain of QDs, a SLED with a semiconductor optical amplifier (SOA) has been integrated [9]. In addition, the multiple contact waveguide device structure has been demonstrated to flexibly select the light emission from GS or ES_1_ of QDs [28].

Although significant progress has been achieved for QD-SLEDs, the spectral emission of QD-SLEDs is limited to the GS or GS + ES_1_ of QDs. Simultaneously introducing GS, ES_1_, and ES_2_ emission in a QD-SLED will definitely further broaden the emission spectrum. However, due to the increased degeneracy of the QDs at high energy transitions, it is very difficult to have all three states’ emission at a comparable power level. In this work, multiple five-QD-layer structures with large dot size inhomogeneous distribution were grown by optimizing the molecular beam epitaxy (MBE) growth conditions. Based on that, by carefully controlling the device’s cavity mirror coating process to accurately control the cavity mirror loss of GS, ES_1_, and ES_2_ of the QDs, respectively, a broadband QD-SLED with simultaneous GS, ES_1_, and ES_2_ emission has been realized, exhibiting over 90 nm spectral bandwidth at a 1 μm wavelength range.

## 2. Materials and Methods

**Figure 1:**
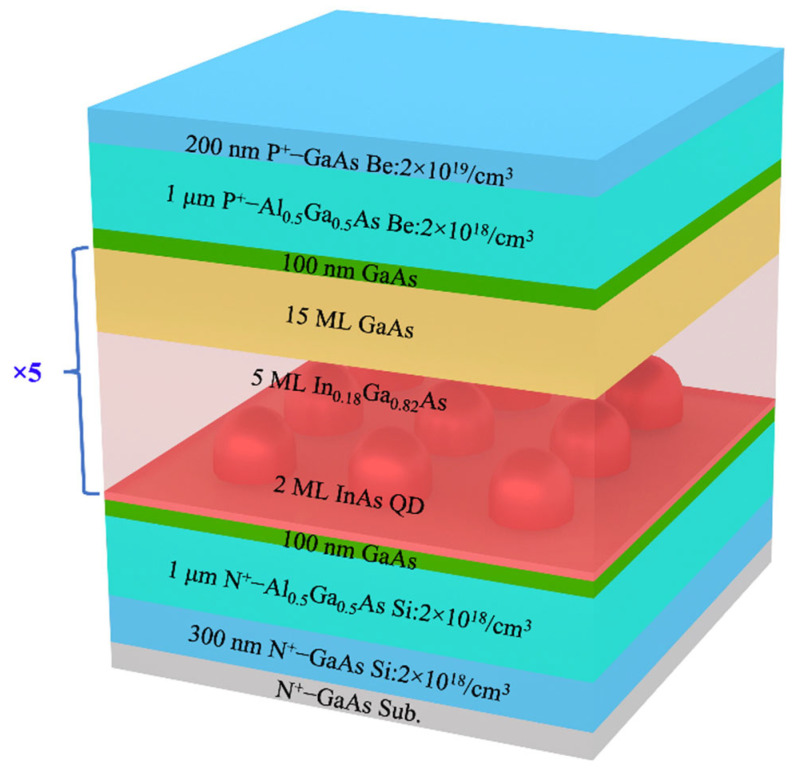
Schematic diagram of the InAs/GaAs QD-SLED structures.

The QD-SLED structure, based on a typical p-i-n configuration, was grown by a solid source MBE reactor on a Si-doped (100) GaAs substrate. Figure 1 schematically illustrates the QD-SLED structure with five stacked InAs QD layers as the active region, whereby each QD layer comprises 2 monolayers (ML) InAs covered with 5 ML In_0_._18_Ga_0_._82_As straining reducing layer (SRL). The whole active region was sandwiched by 1 μm lower n-Al_0_._5_Ga_0_._5_As and 1 μm upper p-Al_0_._5_Ga_0_._5_As cladding layers, and the p^+^-GaAs electrical contact layer completed the epitaxy growth process. The growth temperature for QD layers and SRLs were ~490 °C.

**Figure 2:**
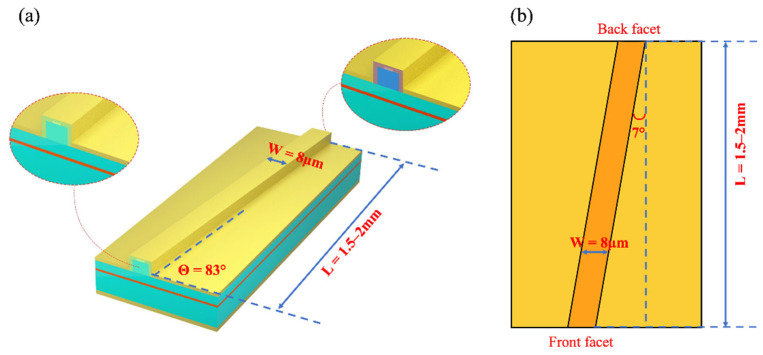
(**a**) Schematic device diagrams (**b**) top view of the tilted waveguide structure.

After the MBE growth, the QD sample was processed into an 8 μm-wide double-trench ridge waveguide structure by standard optical lithography, dry etching, and wet etching techniques, as shown in Figure 2. Then, an insulating SiO_2_ layer was deposited by the plasma-enhanced chemical vapor deposition technique. Electrical contact windows were fabricated at the top of the ridges by photolithography and reactive ion etching. The Ti/Au top metal contact was deposited by electron-beam evaporation. Afterwards, the substrate was thinned down to around 110 μm to minimize self-heating effects, and Ni/AuGe/Ni/Au was deposited on the back side of the wafer. Contacts were alloyed for 60 s at 400 °C. The SLED chips were mounted on indium-plated copper heatsink but without active cooling. The entire characterization was performed at room temperature (RT) under continuous wave (CW) operations.

## 3. Results and Discussion

**Figure 3:**
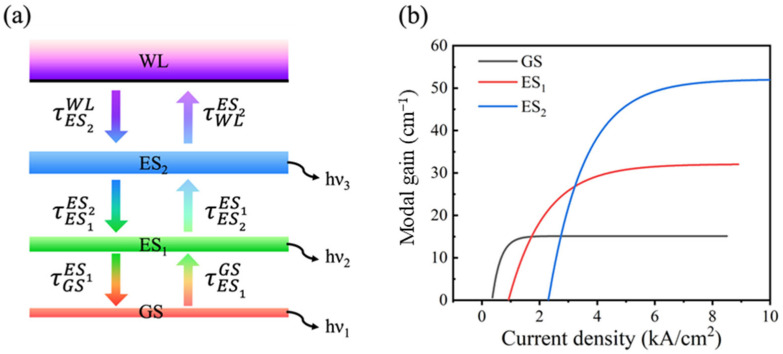
(**a**) Sketch of the energy levels and the carrier dynamics model. The $\tau_{ES_{2}}^{WL}$, $\tau_{ES_{1}}^{ES_{2}}$ and $\tau_{GS}^{ES_{1}}$ are the relaxation time for the carriers, and the $\tau_{WL}^{ES_{2}}$, $\tau_{ES_{2}}^{ES_{1}}$, and $\tau_{ES_{1}}^{GS}$ are the escape time for the carriers. (**b**) A typical modal gain curves of the ground, the first excited, and the second excited levels for QD structures.

Generally, in order to suppress device lasing, various solutions have been proposed, such as tilting the waveguide at an angle to the cleave facets, wet etched facet, anti-reflection coating [28,29], or combinations of the above methods. In this work, as shown in Figure 2a,b, the ridge structure was 7°, inclined with respect to the cavity facets to reduce the reflectivity, and a front facet anti-reflection coating was fabricated by electron beam evaporation. As mentioned above, following the simultaneous emission of the GS and ES_1_ to realize a broadband light source, ES_2_ can be introduced to further broaden the emission spectrum. The three-dimensional confinement of electronic in QDs structure gives rise to a completely discrete energy spectrum, often denoted artificial atoms, the energy level degeneracies of GS, ES_1_, and ES_2_ are 2, 4, and 8, respectively [30]. Therefore, the schematic of the carrier dynamics in a QD-SLED is shown as Figure 3a, and the typical modal gain is shown in Figure 3b. The excited states have larger modal gain due to the higher degeneracy at higher QD energy levels [30,31]. The external injection carriers are assumed to directly fill the WL and then be captured by the dots. Once in the dots, the carriers relax from the higher energy levels to lower energy levels, sequentially from ES_2_ to ES_1_ and to GS. Inversely, carriers can also escape the dots through thermal excitation. For these QD devices, increasing degeneracy of the QD transitions with increasing energy results in difficulties in achieving three state emission. The GS lasing and the slow accumulation of carriers in ESs with increasing injection currents are the two main reasons inhibiting the appearance of all three states of QDs at a comparable power level. In order to obtain simultaneous three-state superluminescence, it is necessary to reduce the number of carriers in the ESs to meet the requirements of light amplification while suppressing the GS lasing. Therefore, it is vital to reduce the loss of the high energy level. The low energy levels can still maintain luminous efficiency when high energy levels are stimulating radiation.

**Figure 4:**
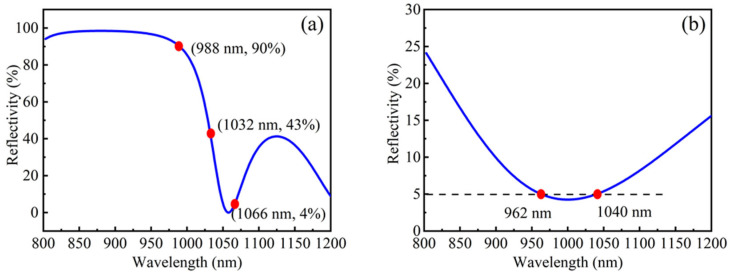
Simulated reflectivity spectra for (**a**) the back facet coating, in which the reflectivity of ES_2,_ ES_1_ and GS are 90%, 43% and 4%, respectively, and (**b**) the front facet coating that the reflectivity of ~5% for all QD emission energy levels.

Facet coating is a key technique for improving the performance of semiconductor photoelectric devices and protecting the damage of facets [31]. In this work, in addition to suppressing the GS lasing of QDs, reducing the mirror loss from ES_1_ and ES_2_ was another critical purpose, so the reflectivity was designed to be high for the wavelength range around ESs of QDs while keeping the reflectivity as low as possible for the wavelength range around GS of QDs. As shown in Figure 4a, six pairs of Ta_2_O_5_/SiO_2_ layers with a central wavelength of λ = 880 nm were designed as the high-reflection (HR) coating on the back facet, in which the reflectivity of ES_2_ and ES_1_ are 90% and 43%, respectively, whereas the GS reflectivity is only 4%, and AR coatings with a reflectivity of ~5% for all QD emission energy levels were applied on the front facet to remove the Fabry-Perot oscillation and to prevent the device lasing.

**Figure 5:**
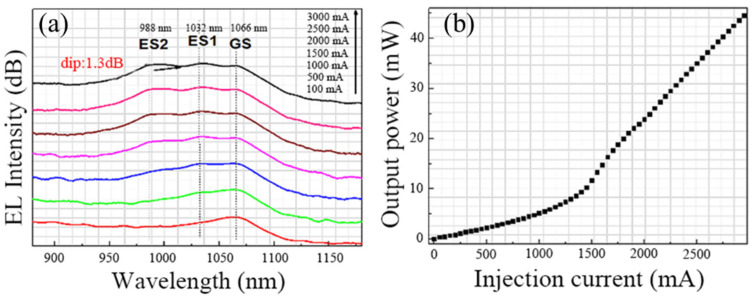
(**a**) EL spectra of QD-SLED at various injection current. (**b**) P-I curve of QD-SLED.

Figure 5a shows the device electroluminescence (EL) spectra against injection currents for the QD-SLED based on the designed facet coating process. Only one peak at ~1066 nm can be observed at a drive current of 100 mA. With an increase in the drive current from 100 to 500 mA, the second peak origin from the ES_1_ at 1032 nm appears, and the GS power continues to increase. Further increasing the injection currents, whereas emissions are maintained from the GS and ES_1_, the third EL peak can be observed at 988 nm, which is attributed to the emission of ES_2_ from the QDs and the 3 dB bandwidth reaching 91 nm. The main factor for this is the long relaxation time from the ES_2_ to ES_1_ and ES_1_ to GS. More importantly, the facet coating design allows the stimulated radiation condition to be satisfied at low carrier populations for ES levels. Hence, the carriers are accumulated in GS, ES_1_, and ES_2_, respectively, leading to the simultaneous three-state emission. Researchers have observed three-state lasing for QDs, but to the best of our knowledge, this is the first realization of simultaneous three-state superluminesence for the QDs based on low incoherent light sources. Furthermore, when the ES_2_ start to emmit, the other two emission peaks gradually become saturated, which is similar to two-state emission QD-based devices [32]. As shown in Figure 5b, a typical superluminescent power-injection current curve can be observed. The device power can be up to 40 mW with the increase in injection currents. The high CW output power in the QD-SLED has further confirmed the advantages of the simultaneous contribution from all three-states of QDs compare to those devices with only GS or GS + ES_1_ emission. In addition, due to the ES_2_ having a large modal gain deriving from double degeneracy, the slope efficiency becomes larger than two-state emission.

**Figure 6:**
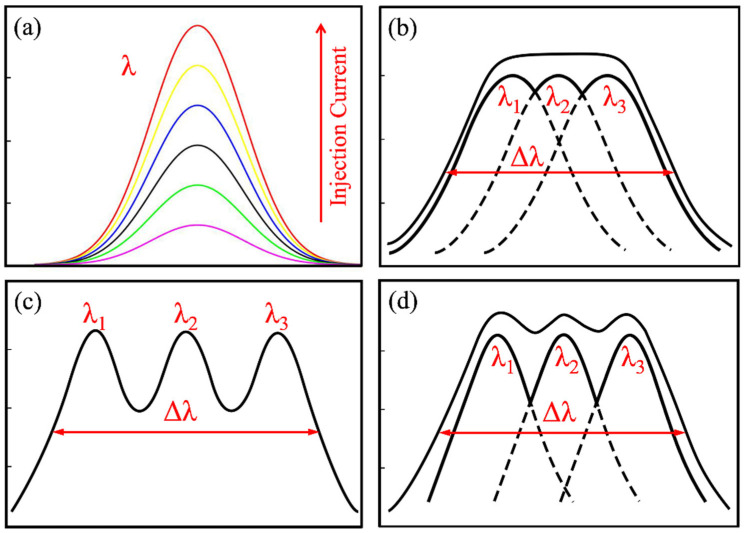
The shapes of the emission spectra of QD-SLEDs. (**a**) Single Gaussian type; (**b**) flat emission spectral type consists of multiple Gaussian spectra; (**c**) multiple-level emission spectral shapes with a large spectral dip; (**d**) multiple-level emission spectral shapes with a small spectral dip. (Δλ is the full width at half-maximum of the emission spectra.).

For many applications, such as OCT systems, in addition to a broad bandwidth and high power, the spectral shape is also very important. The signal detected from a single reflection plane for low-coherence imaging is given by the self-coherence function. Figure 6 shows a heuristic illustration of the influence of the luminescence spectral band for OCT. This function is given by the inverse Fourier transform of the power spectral density of the source and can be regarded as the point-spread function of the imaging system [33]. Therefore, as shown in Figure 6a,b, a single Gaussian and a flat-topped emission spectrum can meet the accuracy requirements of OCT. However, as shown in Figure 6c, the large spectral dips between the different emission peaks will result in side lobes in the point-spread function, which results in an increase in noise floor values at best, and in the creation of ghost images in OCT images at worst. The requirement for bandwidth, power, center wavelength, and controlled spectral shape is therefore highlighted. For QD-SLED, wide energy separation can effectively increase the full width at half-maximum of the emission. However, the large energy separation of λ_2_−λ_1_ or λ_3_−λ_2_ could easily result in larger spectral dips as well. In order to reduce the spectral dips, increasing the inhomogeneous dot size distribution is crucial because the relatively wide emission spectra from each individual λ_1_, λ_2_, λ_3_ emission have more overlaps that can be used to form a flat emission spectrum as shown in Figure 6d. In this work, by optimizing epitaxial growth parameters, especially using the lower growth temperature of QDs and with assistance of the designed facet coating, the spectral dip is only 1.3 dB, which is beneficial for OCT imaging applications.

## 4. Conclusions

In this work, a multiple InAs/GaAs QD-layer structure with large inhomogeneous dot size distribution has been grown by controlling the molecular beam epitaxy growth parameters. Based on that, a tilted stripe waveguide structure SLED with designed facet coating has been fabricated, in which the cavity mirror loss of GS, ES_1_, and ES_2_ of QDs can be accurately controlled, respectively. A broadband and high-power QD-SLED with simultaneous GS, ES_1_, and ES_2_ emission with small dip of 1.3 dB has been realized, exhibiting a high RT-CW output power of 40 mW and over 90 nm broad bandwidth.

## Data Availability

The data presented in this study are available on request from the corresponding author.
